# AGE-FRIENDLY CAMPUSES: NEED AND OPPORTUNITY FOR SYNERGY IN THE AGE-FRIENDLY ECOSYSTEM

**DOI:** 10.1093/geroni/igad104.0335

**Published:** 2023-12-21

**Authors:** Joann Montepare

**Affiliations:** Lasell University, Newton, Massachusetts, United States

## Abstract

Historic shifts in age demographics are reshaping societies and challenging institutions of higher education to respond to older, more age diverse populations through new approaches to teaching, research, and community engagement. Student populations are also becoming more age diverse with adults looking to higher education for lifelong learning opportunities to enhance their personal and professional development as they age. This presentation will describe the guiding framework and set of principles provided by the Age-Friendly University (AFU) initiative to help institutions of higher education shape more age-inclusive practices. Age-friendly campuses can also create opportunities to support other initiatives beyond a campus such as those geared toward age-friendly communities, health care systems, and employers. This presentation will also discuss how synergistic AFU partnerships with other age-friendly initiatives can provide mutual support to strengthen and sustain these efforts. Challenges to this end will also be explored. Examples of existing and promising connections in the ecosystem will be offered to demonstrate the value of this synergy.

